# Cytomegalovirus and Epstein-Barr Infections: Prevalence and Impact on Patients with Hematological Diseases

**DOI:** 10.1155/2020/1627824

**Published:** 2020-10-24

**Authors:** Jean de Melo Silva, Renato Pinheiro-Silva, Anamika Dhyani, Gemilson Soares Pontes

**Affiliations:** ^1^Programa de Pós-graduação em Hematologia, Universidade do Estado do Amazonas, Manaus-Amazonas, Brazil; ^2^Laboratório de Virologia e Imunologia, Coordenação Sociedade, Ambiente e Saúde, Instituto Nacional de Pesquisas da Amazônia (INPA), Manaus-Amazonas, Brazil

## Abstract

Cytomegalovirus (CMV) and Epstein-Barr virus (EBV) infections are widely distributed throughout the world. EBV is linked to various hematological and autoimmune disorders whereas CMV might play important role in the progression of chronic hematological diseases, such as hemoglobinopathies, lymphomas, myelomas, hemophilia, and aplastic and sickle cell anemia. Both viruses produce a viral homolog of human interleukin-10 that can cause general suppression of immune response, increasing susceptibility to other infections. These viruses can remain latent in the host cells and be reactivated when the host immune system is compromised. Studies showing the impact of CMV and EBV infections on hematological disorders are scarce and unclear in the context of coinfection. This review intends to present the biology, prevalence, and impact of CMV and EBV infections in patients with hematological diseases.

## 1. Introduction

CMV and EBV prevalence range from 60% to 100% of the global population, with wide variation between developed and developing countries. Western Europe and the United States generally have the lowest rates, while in South America, Asia, and Africa, (especially in Africa sub-Saharan), the prevalence can reach 100% among adults [1–5]. These two viruses can cause infectious mononucleosis that is mainly characterized by fever, pharyngitis, and lymphadenopathy [6]. After primary infection, EBV and CMV can establish lifelong persistent infection in the host through the latent state, which may be reverted into productive infection under circumstances of immunosuppression [7].

CMV and EBV are highly opportunistic and, therefore, a common cause of infectious disease in immunocompromised patients, such as HIV-infected individuals and organ transplant recipients [8]. Both viruses can establish latency in the host cell, which represents a risk for the quality, safety, and efficiency requirements of transfusions or transplantation, considering that CMV can keep persistence in leukocytes and EBV in B lymphocytes [9]. In this regard, the best alternatives to avoid transfusion or transplantation transmitted infection are the leukoreduction of blood products and the serological screening of potential donors [10]. Besides, it is very important to understand how the immunocompromised patients are affected by transfusion-transmitted CMV/EBV infection, once these individuals are at increased risk of developing severe CMV disease.

CMV and EBV infections are also a common cause of complications in patients with hematological malignancies and one of the major limiting factors in the successful treatment of patients [11]. However, there are very few studies reporting EBV/CMV prevalence among patients with hematological diseases. Moreover, the impact of these infections in the clinical course of hematological diseases is also little understood. Most of the studies are related to organ transplant patients, individuals with HIV, and pregnant women. Thus, this review aims to provide an overview of the current knowledge concerning the prevalence of these infections and their impact on patients with hematological diseases.

## 2. Methodology

We performed a literature review focusing on both qualitative and quantitative studies to address the key biological and clinical aspects of CMV/EBV infection, in the context of hematological diseases. The databases assessed were PubMed, ScienceDirect, and SciELO. The terms used in the search were Cytomegalovirus, Epstein-Barr virus, CMV, EBV, hematological diseases, hematological malignancies, prevalence, leukemia, lymphoma, infectious mononucleosis, coinfection, blood, reactivation, acute infection, and anemia. A total of 500 articles came up from the search strategy used.

Then, these articles were screened based on studies within a relevance to the review purpose, following a scoping review protocol. The screening resulted in 200 articles. From this number, 114 articles were selected to be included in this review based on a refined analysis done by our research group. Systematic reviews, meta-analysis, and case reports were also included. The search was undertaken from 15 January to 18 June 2020.

## 3. Viral Structure of CMV and EBV

**Figure 1:**
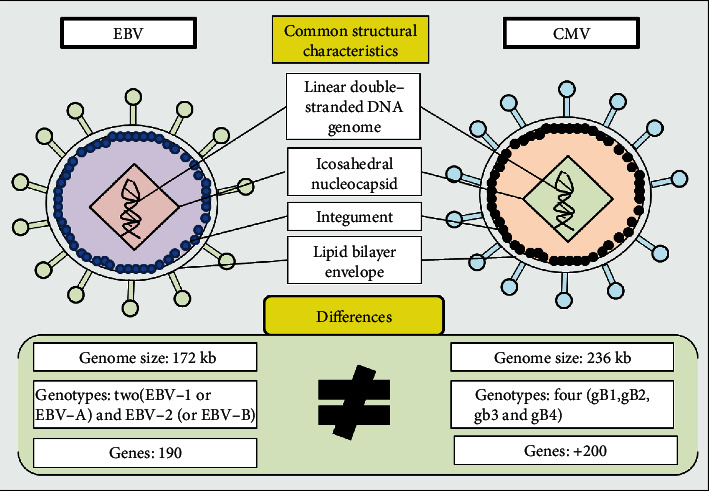
Structural characteristics of CMV and EBV.

CMV and EBV belong to the family *Herpesviridae*, subfamilies *Betaherpesvirinae*, and *Gammaherpesvirinae*, respectively [12]. As members of the herpes virus family, these viruses share common structural characteristics [13, 14, 16–19]. The complete virus particles are composed of a large linear double-stranded DNA genome, protected by an icosahedral nucleocapsid, which is surrounded by an integument that is consisted of an amorphous layer of viral proteins and viral mRNAs. The viral particles are enclosed by a lipid bilayer envelope [13, 14]. Some structure and life cycle characteristics of CMV and EBV are presented in Table 1 and Figure 1.

Human CMV (species *Human herpesvirus 5*) has the largest genome of any known human virus. The size of the spherical particle ranges from 200 to 300 nm, and its 236 kbp genome is constituted by unique long (UL) and unique short (US) regions, both flanked by inverted sequences. The CMV genome has 158 open reading frames (ORFs) encoding 41 essential and 117 nonessential proteins for CMV replication [20]. The icosahedral nucleocapsid is around 110 nm, composed of four integral proteins (pUL46, pUL80.5, pUL85, and pUL104), which are organized into 162 capsomeres (150 hexamers plus 12 pentamers) with 320 triplexes between the capsomeres [21]. The CMV tegument comprises approximately 38 viral proteins essential for initiation of virus replication and several cellular proteins and mRNAs with unknown function [22]. The tegument layer is enclosed by a lipid envelope predominantly composed of the glycoproteins B (gB), gM, gN, gH, gL, and gO. These proteins are the major targets of immune responses and play a key role in the attachment and penetration of the virus into the host cell [23, 24].

The categorization of CMV genotypes is made based on the glycoprotein sequences. The genotypes gB (gB1-gB4) and gN (gN1-gN4) are originated from the sequence diversity of the glycoprotein B (UL55 gene) and glycoprotein gN (UL73 gene), respectively [25]. Genotype gN3 is subdivided into gN-3a and gN-3b, and the genotype gN-4 is subdivided into gN-4a, gN-4b, and gN-4c. The diversity of the gH glycoprotein also results in two genotypes (gH1 and gH2) [26]. The EBV particle encompasses a toroid shaped protein core wrapped with DNA, a nucleocapsid with 162 capsomeres, a protein tegument consisting of viral and cellular proteins, and an outer lipid envelope layer with external virus-encoded glycoprotein spikes. The EBV particle comprises a toroid shaped protein core wrapped with DNA, a nucleocapsid with 162 capsomeres, a protein tegument consisting of viral and cellular proteins, and an outer lipid envelope layer with external virus-encoded glycoprotein spikes [27].

## 4. Life Cycle of CMV and EBV

**Figure 2:**
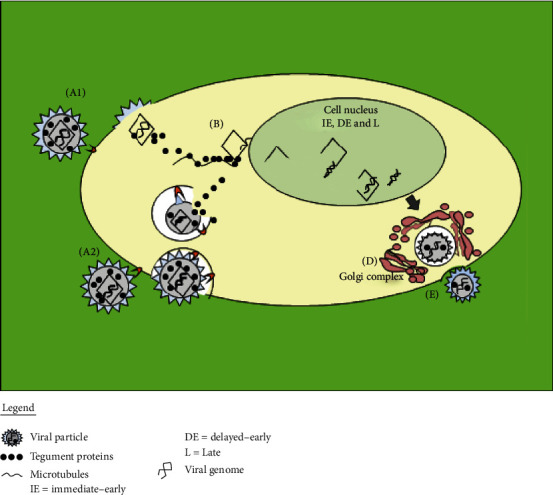
CMV and EBV life cycles. (a1, a2) Viral invasion. (b) Capsid transportation to the nucleus. (c) Expression of IE, DE, and L genes and start of genome replication. (d) Formation of the viral assembly complex (AC). (e) Release of the new infectious particles.

CMV and EBV are nuclear*-*replicating viruses with a life cycle consisted of cellular invasion, replication, and latency. During the cellular invasion (Figure 2), glycoproteins of the viral envelope interact with host receptors, leading to fusion (Figure 2(a1)) or endocytosis (Figure 2(a2)). Once inside of the host cells, the viral tegument proteins interact with microtubules of the cytoplasm (Figure 2(b)). This interaction facilitates the transport of capsids to the nucleus, where the transcription, genome replication, and capsid assembly take place (Figure 2(c)) [28].

Viral genome replication is divided into three phases: immediate-early (IE) genes, which encode regulatory proteins; delayed early (DE) genes, which encode enzymes for replicating viral DNA; and late genes (L), which encode structural proteins. Once the viral capsid is assembled, it buds out of the nucleus by disrupting the nuclear membrane and getting associated with tegument proteins to form new infectious particles (Figure 2(d)). These new particles are transported to the cell membrane via the Golgi complex and released into the extracellular space (Figure 2(e)). Alternatively, depending upon the type of host cells, the virus can establish latency. The latent CMV genome may be reactivated at any time, especially when the host immune system is compromised. The myeloid lineage cells and CD34+ progenitor cells are an important site of CMV latency [29, 30]. CMV reactivation poses a risk to the health of the immunocompromised patients (such as solid organ and stem cell transplant recipients), which can lead to organ damage, among other clinical manifestations [31].

Unlike CMV, the latency of EBV is subdivided in 0, I, II, and III phases. Latency type 0 occurs inside of memory B-cells, where EBV remains undetectable to the immune system. Latency type I refers to the expression of Epstein-Barr nuclear antigen 1 (EBNA-1) and Epstein–Barr virus-encoded small RNAs **(**EBERs), both required for the division of the viral genome. During the latency type II, aside from EBNA-1 and EBERs, latent membrane protein (LMP)-1, LMP-2A, and LMP-2B genes are also expressed to induce the proliferation and transformation of the host cells. In latency type III, proteins are expressed to immortalize the infected cells [32].

EBV reactivation has three lytic phases: immediate-early (IE), early (E), and late (L). In the IE period, the BZLF1 gene encodes the Zta transactivator and the *BRLF1* gene encodes the Rta transactivator. In the early period, genes involved in the EBV replication are expressed. The expression of late genes happens after the EBV DNA replication. During this phase, structural proteins (capsid and membrane proteins) are encoded to lead the genome encapsidation and, consequently, the production of new viral particles [33].

Like any other virus, CMV and EBV cycle may dictate the pathogenic mechanisms that shape their correlated diseases. Thus, understanding the interaction between CMV or EBV and its host is crucial to uncover all aspects that encompass the disease processes produced by infection. Unfortunately, studies that aim to understand the progression of CMV/EBV infection, from virus entry through spread to release of virus progeny, are rare. This strategy is crucial to unveil the mechanisms of virus pathogenesis, clearance, and persistence.

## 5. Clinical Manifestations of CMV and EBV Infections

CMV or EBV primary infection is usually asymptomatic in adults and children. In immunocompromised individuals, acute CMV infection may cause oral ulcers, periodontitis, sialadenitis, gingival hyperplasia, esophagitis, black esophagus, hepatitis, pancreatitis, gastritis, appendicitis, and among other clinical manifestations [34, 35]. In immunocompetent individuals, the usual clinical symptoms include pharyngitis, lymphadenopathy, fever, and mononucleosis-like syndrome [36]. CMV shows tropism for salivary glands and can infect different cell types, including endothelial, epithelial, muscle, and fibroblast cells [37, 38]. CMV infection may cause cytopathic effect characterized by nuclear and cytoplasmic inclusions, resulting in morphological changes that make the infected cell hypertrophic [29]. In HIV patients, CMV infection can cause retinitis (in 80% of cases), gastrointestinal manifestations (in 10 to 15% of cases), colitis, esophagitis, hepatitis, encephalitis, myeloradiculopathy, and lung involvement (1-5% of cases) [39]. Moreover, both CMV and EBV are frequently detected in the intestinal mucosa of HIV patients and might lead to local and systemic inflammation [40].

CMV infection might play an important role in the progression of various chronic hematological diseases, such as hemoglobinopathies, lymphomas, myelomas, hemophilia, and aplastic and sickle cell anemia. In individuals suffering from these conditions, CMV infection may also affect the gastrointestinal tract (causing colitis), lungs (causing pneumonia), and central nervous system (causing transverse myelitis, encephalitis, and meningitis) [41, 42]. Besides, CMV can decrease platelet production, because it can infect directly the megakaryocytes [43]. Evidence indicates that CMV can also protect tumor cells from the apoptosis mechanism, favoring the resistance of these cells to chemotherapy treatment [44].

In the case of EBV, oral pharynx epithelial cells are the local where primary EBV infection is set out, but EBV establishes persistent latent infection in B cells [45]. Infected B cells multiply excessively when EBV forms an episome in the nucleus, which guides to cell transformation [46]. EBV has been implicated in many autoimmune, neurological, and hematological diseases as well as other malignancies, such as Hodgkin's lymphoma, Burkitt's lymphoma, gastric carcinoma, posttransplant lymphoproliferative disease, and nasopharyngeal carcinoma [47, 48]. However, the main clinical manifestation resulted from EBV primary infection is the infectious mononucleosis (IM), observed especially in young children and teenagers [49, 50]. IM is an acute febrile disease characterized by fever, pharyngitis, malaise, lymphadenomegaly, and atypical lymphocytosis [51, 52]. In poor countries, EBV infection occurs predominantly during childhood. Notwithstanding, in developed nations, it affects individuals between 10 and 30 years old [2]. IM can lead to hematologic disorders, including prolonged hemolytic anemia and thrombocytopenia [53].

Healthy people can develop Chronic Active EBV Infection (CAEBV), a disease characterized by persistent EBV infection. In this scenario, infected individuals present mononucleosis-like symptoms and high EBV loads in the peripheral blood [54]. Exacerbated inflammatory response due to the increased proinflammatory cytokine secretion (macrophage colony-stimulating factor, IL-6, IL-10, TNF-alpha, IFN-gamma, and others) is responsible for the severe CAEBV infection [55]. CAEBV patients usually present fever, splenomegaly, and liver dysfunction. Other manifestations can include anemia, thrombocytopenia, lymphadenopathy, rash, hemophagocytic syndrome, coronary artery aneurysms, mosquito bite hypersensitivity, central nervous system disease, basal ganglia calcification, oral ulcers, and interstitial pneumonia and lymphoma [56].

In HIV-infected individuals, EBV infection can cause atypical lymphoproliferation, lymphadenopathy, and lymphoid malignant transformations as an outcome of proinflammatory cytokine stimulation, such as tumor necrosis factor (TNF) [57]. Stevens et al. (2002) [58] found EBV DNA in the peripheral blood of patients receiving Highly Active Antiretroviral Therapy (HAART) (64/109 samples), and 34% of these patients presented EBV DNA loads above 2.000-89.400 copies/ml blood.

Viral coinfection among immunocompromised patients is often observed. A study performed in Amazon, Brazil, verified that anal cancer in HIV-infected patients was associated with EBV/CMV infection. The coinfection was detected in 16.9% of patients with anal cancer [59]. Aalto et al. (1998) [60] reported that CMV infection creates a favorable environment for EBV reactivation in the context of coinfection, since it increases the immunosuppressive status [61].

Another study detected EBV and CMV coinfection in pediatric patients with leukemia. The researchers noted a borderline association with worse overall survival, indicating that EBV and CMV infections may act upon the prognosis and the survival rates of these patients [62]. In HIV-1 seropositive patients from India, Patekar et al. (2015) [63] ascertained that patients with high antibody titers against EBV/CMV exhibited the lower T cell CD4+ counts (less than 200 cells/mm^3^). Although the pathogenic mechanisms are so far unclear, these studies clearly show the potential risk of CMV and EBV infection (or coinfection) in the context of immunosuppression, a situation that could result in the development of severe clinical manifestations.

## 6. Prevalence of CMV and EBV Infections in Patients with Hematological Diseases

Even with high morbidity linked to CMV and EBV infections, there are very few studies investigating the prevalence of these viruses in patients with hematological diseases.  Table 2 summarizes most of these studies.

Piukovics et al. (2017) [64] retrospectively (2008-2014) investigated the incidence of CMV infection in a total of 271 patients with malignant hematological diseases. The majority of the patients (82.6%) were suffering from lymphoproliferative disorders, like multiple myeloma (MM), lymphomas, lymphocytic or lymphoblastic leukemia, and myeloid malignancies (acute myeloid leukemia, myelodysplastic syndrome). The authors reported the presence of CMV IgG antibodies in 154 patients (75.5%). From this number, 66 (24.4%) patients were positive for CMV DNA. The highest rate of CMV infection (33.3%) was observed in patients who had received autologous stem cell transplantation.

Loutfy et al. (2017) [62] found EBV or CMV DNA in 56% of pediatric patients with leukemia, who showed an EBV/CMV coinfection rate of 18%. Nevertheless, as the number of EBV/CMV coinfected patients was small, no statistical association between coinfection and leukemia severity was found. The distribution of CMV DNA in serum and leukocytes were 36% and 20%, respectively. On the other hand, EBV DNA frequencies were 2% in serum and 28% in leukocytes.

A serological study performed in the city of Yazd (one of the most populous urban centers in Iran) by Moghimi et al. (2015) [65] verified the presence of IgM and IgG antibodies against CMV in patients with beta-thalassemia major. From a total of 96 patients evaluated, 94.1% showed positivity for IgG and 10% for anti-CMV IgM antibodies. Higher CMV seropositivity (71.4% for IgG and 60% for IgM) was noticed in patients who have undergone blood transfusions regularly (average of 20 days interval between the transfusions). This data demonstrates the imminent risk of CMV infection be acquired through blood transfusion.

Slavov et al. (2015) [66] investigated the presence of CMV DNA in patients suffering from sickle cell disease and beta-thalassemia major attended at the Regional Blood Center of Ribeirão Preto, Brazil. They observed a prevalence of 13.8% in patients with sickle cell disease and 7.6% in patients with beta-thalassemia major. The CMV genotype gB2 was more prevalent (90.9%) in comparison with the gB1 genotype (9.1%). This study highlights the importance of genotype identification during clinical management, especially to understand the role of CMV infection in the context of hematologic disease.

A study carried out in a blood bank of Bahia state (Brazil) verified a seroprevalence of CMV in 89.4% of patients with hematological diseases (*n* = 470). In patients carrying “other type of anemia,” the prevalence observed was 78.9%, and among individuals suffering from other hematological disorders, such as sickle cell anemia, cancer, hemophilia, and hemoglobinopathies, the prevalence was 85% [41]. A high frequency of active CMV infection has also been reported in hemophiliac patients. Nogueira et al. (2000) [67] observed the presence of CMV DNA in 25% (25/100) of patients with hemophilia and most of them presented gastrointestinal bleeding possibly correlated with CMV infection.

Few studies have reported the EBV prevalence among patients with hematological diseases. In Croatia, seroprevalence rates of 91.2% and 97.7% were found among patients with hematological disorders and patients submitted to hemodialysis, respectively [68]. Guan et al. (2017) [69] studied the correlation between EBV infection and acute leukemia. They reported EBV prevalence rates of 40.9% and 25.3% in patients with acute lymphoblastic leukemia (ALL) and acute myeloid leukemia (AML), respectively. The authors suggested a possible correlation between EBV infection and the development of acute leukemia (unfavorable prognosis was also observed in EBV infected patients). Another study recorded the presence of EBV DNA in 68.5% of patients suffering from B-cell non-Hodgkin Lymphomas (NHL) [70]. Ruskova et al. (2004) [71] showed five cases of aggressive natural killer-cell leukemia (ANKL) in patients seropositive for IgG anti-EBV.

Clearly, CMV or EVB infection is highly prevalent among patients suffering from hematological diseases throughout the globe and these infections can influence the patient prognostic. However, the mechanisms by which these viruses increase morbidity or mortality of patients with hematological diseases are still a matter of debate.

## 7. CMV and EBV Infection Impact in Patients with Hematological Diseases

**Figure 3:**
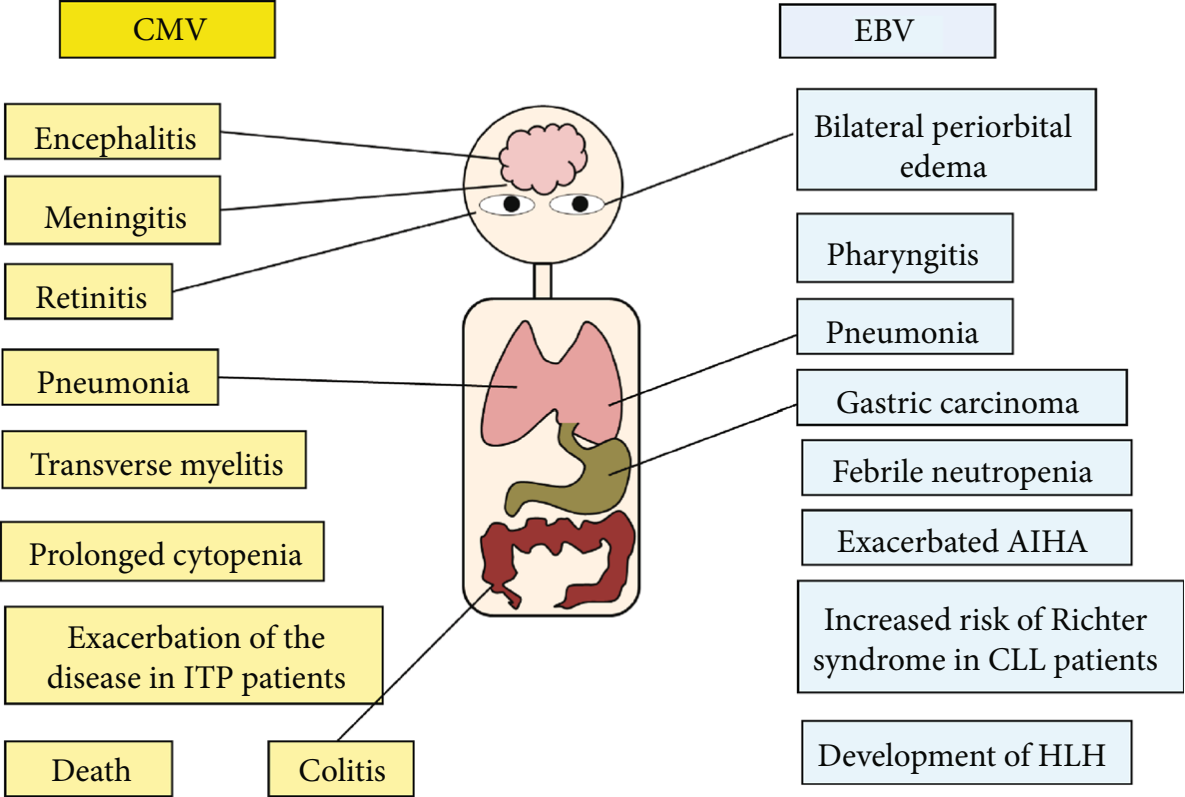
Clinical manifestations associated to CMV or EBV infections in patients with hematological diseases.

CMV infection is the major cause of morbidity and mortality in patients with hematological diseases, whereas EBV infection has been mostly described in myeloma, leukemia, and lymphoma patients with high-risk factors posttransplant [64, 72–74]. The main clinical manifestations caused by CMV and EBV infections in patients with hematological diseases are summarized in Figure 3. About 25 to 50% of EBV infectious mononucleosis may result in hematologic complications, such as hemolytic–uremic syndrome, thrombotic thrombocytopenic purpura (TTP), disseminated intravascular coagulation (DIC), hemolytic anemia, aplastic anemia, or thrombocytopenia [6, 75].

Few studies are available on the true impact of CMV infection upon hematological diseases. Most of the studies related to CMV infection were conducted in individuals with a high risk of developing severe clinical manifestations, such as HIV/AIDS carriers, pregnant women, newborns, hemodialysis patients, transplanted patients, and immunocompromised or immunosuppressed individuals [76–81]. Nonetheless, CMV infection can be a serious threat for patients with hematological diseases receiving chemotherapy treatment [82]. It was verified a CMV reactivation rate of 15.3% in patients with ALL [83]. Normally, higher susceptibility to viral reactivations has been observed in ALL patients than in patients with AML, probably because of the chemotherapy deleterious effect on the lymphocyte cell population [84].

Zhou et al. (2014) [85] verified that CMV reactivation is one of the main factors associated with febrile neutropenia in patients with hematological diseases. Indeed, an increased risk of CMV infection/reactivation in patients with hematological diseases has been observed, especially among those under nontransplant treatment [86]. CMV and EBV reactivations occur frequently in patients with Adult T Cell leukemia (ATL) [87]. Nguyen et al. (2001) [88] reported a low but increasing prevalence of CMV pneumonitis in patients with leukemia between 1992 and 1997 (prevalence doubled from 1.4% to 2.8% in 5 years). The mortality rate caused by CMV-associated pneumonia was 57%. Deaths occurred about 15 days after the onset of pneumonia, revealing that CMV is an important cause of pneumonia in adults with leukemia, particularly on those undergoing immunosuppressive therapy.

Children with NHL or ALL having prolonged cytopenia showed a prevalence of 54% for CMV infection, suggesting the virus infection as a potential cause of prolonged cytopenia after conventional chemotherapy [89]. This negative clinical impact elicited by CMV infection reveals the importance of CMV epidemiological investigation in patients with hematologic disease and the need for studies that open up the CMV mechanisms linked to clinical manifestations, such as cytopenia. Kennedy-Nasser et al. (2008) [90] reported that relapses viral infections (especially CMV infection) were the primary cause of death in a group of 83 children with ALL. Ozkale et al. (2015) [91] described a case of intractable diarrhea due to colitis caused by CMV infection in an immunocompromised adolescent with hereditary spherocytosis. This patient presented acute jaundice, abdominal pain, cholelithiasis, choledochal stones, and dilated proximally bile ducts sepsis. A liver biopsy also revealed a chronic cholestatic hepatitis.

Ding et al. (2007) [92] conducted a study in China to diagnose the CMV infection in 81 children with immune thrombocytopenic purpura (ITP). They observed that ITP patients with CMV infection were more likely to have a serious clinical manifestation of ITP, besides being refractory and presenting ITP chronicity. This data suggest that CMV infection might be an important risk factor for the occurrence of severe and persistent ITP during childhood [92].

It is already known that blood transfusion is an important risk factor for CMV infection, since leukocytes are important persistent sites of latent CMV. However, although the majority of individuals who acquire CMV through transfusion do not present symptoms, some of them may show some variable clinical manifestations that can occasionally evolve and lead to death in an immunosuppression scenario [41, 93].

EBV infection is also associated with the development or prognosis of different hematological diseases. Hamilton et al. (2015) [94] reported an autoimmune hemolytic anemia case associated with EBV infection. The presence of IgG autoantibody against erythrocytes was confirmed along with the high EBV viremia in a renal transplant recipient, five years after kidney transplantation. Another case-control study observed a high prevalence of EBV-associated gastric carcinoma in patients with pernicious anemia [95]. Fadeyi et al. (2015) [96] reported a case of fatal autoimmune hemolytic anemia (AIHA) associated with the reactivation of latent EBV infection in a 31-year-old man. The authors suggested that EBV infection triggered severe hemolysis, which aggravated the AIHA. Similarly, EBV infection reactivation impacted the prognosis of AIHA in a patient presenting high titers of serum EBV DNA [97].

An observational study carried out in India with 120 patients suffering from aplastic anemia found active EBV infection in 20% of the patients. From this number, seventeen individuals showed severe, five very severe, and two nonsevere aplastic anemia. None EBV IgM positive patients had mononucleosis at the time of laboratory diagnosis. Two patients positive for EBV and coinfected with parvovirus infection had severe aplastic anemia [98].

EBV DNA infection has been also associated with the pathogenesis of ALL, but the mechanism is still unclear [99]. A recent study identified the presence of EBV DNA in 43% of individuals suffering from different hematological diseases, including Hodgkin lymphoma, non-Hodgkin lymphoma, Chronic Lymphoid Leukemia (CLL), and ALL [100].

A case reported by Sung et al. (2007) [101] suggested for the first time the EBV latent infection as a cause of pure red cell aplasia (PRCA) in a patient with NHL. The common cause of PRCA is a viral infection and the human parvovirus B19 (B19V) is the main etiological agent. However, in this study, the authors reported the expression of EBERs *antigens* in a patient's bone marrow cells, suggesting the latent EBV infection as a possible cause of PRCA. This study demonstrated the importance of investigating the EBV infection in patients with PRCA [101]. Also, EBV or CMV-infected patients with PRCA, who received antiviral and immunosuppressive drugs responded positively to this therapeutic strategy [102]. These data show the importance of including antiviral medication in the therapeutic management of PRCA in the context of EBV or CMV infection. Antiviral therapeutic strategy in this context is also motivated by the fact that the EBV-associated chronic PRCA can be related to T-cell mediated suppression of erythroid colony-forming (ECF) unit proliferation in the bone marrow [101, 102].

An aggressive lymphoma known as Richter syndrome (RS) that usually arises in CLL patients might be associated with EBV reactivation [103, 104]. In RS, the large cells may represent a new neoplasm or they could emerge through the transformation of the original CLL clone, which might occur due to the EBV reactivation [105]. The association between EBV reactivation and the increased risk of RS development in CLL patients during immunosuppressive therapy was also reported [106]. The pathogenesis and course of CLL condition have been linked to EBV-induced STAT-5, which may be associated with the rise of the mortality rates among CLL carriers [107].

EBV-associated uncontrolled cell proliferation in immunocompromised individuals commonly leads to increased emergence of lymphoproliferative disorders [32]. Not only reactivation but also primary infection may cause severe damage in these individuals.  Table 3 shows case reports of EBV acute infection or reactivation in patients suffering from hematological diseases. Potenza et al. (2007) [108] reported the first case of pneumonia linked to EBV reactivation in an adult patient with severe aplastic anemia (SAA). Lu et al. (2016) [109] presented two cases of patients with acute lymphoblastic leukemia showing EBV-related clinical manifestations. Patient 1 was a 16-year-old male diagnosed with EBV acute infection (viral load of 63.700 copies/mL) that developed febrile neutropenia and pharyngitis EBV acute infection. Patient 2 was a 12-year-old male positive for IgG against EBV (viral load of 101.000 copies/mL) who presented a 38.6°C fever and progressive bilateral periorbital edema. Nonetheless, it was not possible to subclassify EBV-positive lymphoproliferative disorders in both cases.

EBV-associated mucocutaneous ulcer (EBV-MCU) was found in a 29-year-old male patient with T-cell acute lymphoblastic leukemia. A biopsy of the EBV-MCU revealed the presence of histiocytes, plasma cells, neutrophils, and dense polymorphous lymphocytes [110]. Cavallari et al. (2017) [111] presented a case of a CLL patient who had EBV infection reactivation and showed a hemophagocytic lymphohistiocytosis (HLH) after Ibrutinib treatment. The author suggested that EBV reactivation along with CLL-related immunodeficiency may have contributed to the development of HLH.

**Figure 4:**
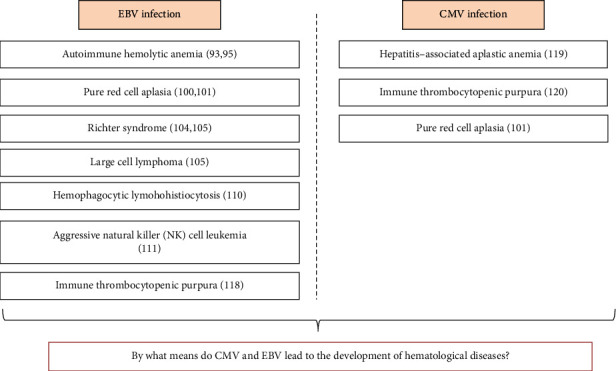
Hematological diseases associated to EBV/CMV infection.

Hu and Wang (2017) [112] reported a case of aggressive natural killer (NK) cell leukemia in a 23-year-old man with no prior medical history. The patient presented high fever for a month, weight loss, dyspnea, nasal bleeding, and EBV serology positive for lgG, but negative for lgM. He died of respiratory failure a week after diagnosis. Another case report from a 71-year-old female diagnosed with EBV-associated lymphoproliferative disorder showed the occurrence of EBV reactivation. The patient showed elevated EBV viral load after treatment with the antitumor necrosis factor [113]. These studies demonstrated that the reactivation of EBV happening in patients with chronic immunologic disorders may be directly linked to the therapeutic scheme (immunomodulatory) they have been through. Thus, EBV reactivation triggered by the treatment might be a prognostic risk factor for the disease worsening in patients with hematological disorders. Recently, Handous et al. (2020) [114] verified that patients suffering from acute leukemia coinfected with CMV/EBV showed the worst prognostic on leukemia treatment, including recurrent CMV/EBV reactivations and complications after chemotherapy.  Figure 4 shows the hematological diseases that can be possibly caused by CMV or EBV infections.

Take all together, CMV/EBV infections are closely related to the worsening prognosis or development of hematological diseases. These infections impose a negative impact on patient morbidity and mortality, especially in low-income countries. However, the viral pathogenic mechanisms underlying the development or worsening of hematological diseases are not well understood.

## 8. Conclusions

The frequency of CMV and EBV infections are elevated in patients with hematological diseases, and both conditions are possibly associated. Although the Burkitt lymphoma is the main hematological disorder related to EBV infection, studies also suggested that the development of different hematological diseases (especially AML and ALL) is linked to EBV infection. In the case of CMV infection, it is mostly associated with the development or poor prognosis of ALL disease.

The risk of CMV infection is interrelated to blood transfusions. However, it is not clear whether blood transfusion increases the risk of EBV infection. Epidemiological surveillance of both infections in patients with hematological diseases is essential to improve their clinical management. Furthermore, the reactivation rates of both viruses are increased among patients with hematological diseases, and it can be related to immunological status imposed by hematological condition.

Nonetheless, many questions regarding the impact of both viruses in the course of hematological diseases remain unsolved, like the following: how to prevent virus reactivation in these patients? Is it possible to improve the prognosis of these patients who are undergoing immunosuppression treatment by submitting them to antiviral therapy? In the case of CMV/EBV coinfection, what are the mechanisms of potential viral cooperation in the production of disease? What are the molecular mechanisms by which the EBV/CMV infections induce clinical manifestations in patients with hematological diseases? These answers can only be provided through studies that especially focus on the dynamics of the viral pathogenesis taking place in these patients.

## Figures and Tables

**Table 1:** CMV and EBV biology.

	Cytomegalovirus	Epstein-Barr virus	Reference
Family, subfamily, and type	*Herpesviridae; Betaherpesvirinae*; HHV-5	*Herpesviridae; Gammaherpesvirinae*; HHV-4	Davison et al. 2005 [15]; Spano et al. 2004 [16]; Bolis et al. 2016 [5]; Santos et al. 2014 [17]; Beltran and Cristea, 2014 [28]; Shabani, Nichols and Rezaei, 2016 [32]; Li et al. 2016 [33]; Landolfo et al. 2003 [18]; Johannsen et al. 2016 [19]; Kalejta, 2008 [22] Mocarski, 2002 [24]; Plachter, Sinzger and Jahn, 1996 [37]; Chen, Jardetzky and Longnecke, 2016 [45]; Oliveira et al. 2012 [2]; Plosa et al. 2012 [38]; Forman et al. 2015 [35]; Hess, 2004 [52];
Genome and genotypes	Linear dsDNA coding more than 200 proteins Four genotypes: gB1, gB2, gB3 and gB4	Linear dsDNA coding for 87 proteins Two genotypes: EBV-1 and EBV-2	
Cycle	Three lytic phases: immediate-early (IE), early (E), and late (L)	Latency types 0, I, II and III^7^ Three lytic phases: immediate-early (IE), early (E), and late (L)	
Capsid proteins	pUL46, pUL48.5, minor capsid protein (mCP), major capsid protein (MCP)	MCP, mCP, mCPBP, sCP	
Envelope proteins	gpUL55 (gB), gpUL73 (gN), gpUL74 (gO), gpUL75 (gH), gpUL100 (gM) e gpUL115 (gL)	gM, gN, gBC, gBN, gB, gp350, gp150, gp42, gL, gH, gp78	
Tegument proteins	38 proteins, including pp28, pp65, pp71, and pp150	LTP, LTPBP, BLRF2, BRRF2, MyrP, MyrPBP, MTP, PalmP, HSP70, BKRF4, BDLF2, and others	
Tropism	Salivary glands, macrophage, leukocytes, and can infect endothelial, epithelial, hematopoietic progenitor, peripheral blood mononuclear, smooth muscle, and fibroblast cells	Oral pharynx epithelial cells Lymphocytes, gastric mucosa, smooth muscle cells, and others	
Transmission	Body fluids (urine, saliva, tears, milk, blood), genital secretions, transplanted organs, and sexual transmission	Body fluids (saliva, blood, and others), transplantation, and sexual transmission	

**Table 2:** Prevalence of CMV or EBV infection in patients with hematological disorders.

Virus	Patient's disease	Country/ City	Cases (n)	Viral prevalence	Reference
CMV	Lymphoid, myeloid hematological malignancies and other hematological diseases	Hungary, Szeged	204	75.5%	Piukovics et al. 2017 [64]
	Leukemia	Egypt, Cairo	50	36.0% (in serum) 20.0% (in WBCs)	Loutfy et al. 2017 [62]
	Thalassemia	Iran, Yazd	96	94.1%	Moghimi et al. 2015 [65]
	Sickle cell disease	Brazil, Ribeirão Preto	144	13.9%	Slavov et al. 2015 [66]
	Beta-thalassemia major		39	7.7%	
	Sickle-cell anemia, cancer, hemophilia, hemoglobinopathy, and other types of anemia	Brazil, Bahia	470	89.4%	De Matos, Meyer and Lima, 2011 [41]
	Hemodialysis patients	Turkey, Antakya	255	99.6%	Ocak et al., 2006 [81]
	Hemophilia	Brazil, São Paulo	100	25%	Nogueira et al. 2000 [67]

EBV	Hematological malignancies	Croatia, Zagreb	103	91.2%	Beader et al. 2018 [68]
	Hemodialysis patients		170	97.7%	
	ALL	China, Qingdao	110	40.9%	Guan et al. 2017 [69]
	AML		75	25.3%	
	Leukemia	Egypt, Cairo	50	2.0% (in serum) 28.0% (in WBCs)	Loutfy et al. 2017 [62]
	Childhood B non-Hodgkin lymphoma	Brazil, Rio de Janeiro	35	68.5%	Klumb et al. 2004 [70]
	ANKL	New Zealand, Auckland	5	100%	Ruskova, Thula and Chan, 2004 [71]

ALL: acute lymphoblastic leukemia; CLL: chronic lymphocytic leukemia; ANKL: aggressive natural killer-cell leukemia.

**Table 3:** Case reports on EBV infection in patients with hematological diseases.

Sexes	Age	Hematological diseases	EBV	Coinfection	Infection type	Survival	Reference
Male	26	Severe aplastic anemia	Yes	No	Reactivation	Alive	Potenza et al. 2007 [108]
Male	16	ALL	Yes	No	Primary infection (acute)	Alive	Lu et al. 2016 [109]
Male	12	ALL	Yes	No	Reactivation	Alive	Lu et al. 2016 [109]
Male	29	ALL	Yes	No	Reactivation	Alive	Vatsayan et al. 2016 [110]
Male	70	CLL	Yes	No	Reactivation	Dead	Cavallari et al. 2017 [111]
Female	71	LPD	Yes	No	Reactivation	Alive	Febres-Aldana et al. 2020 [113]

ALL: acute lymphoblastic leukemia; CLL: chronic lymphocytic leukemia; LPD: lymphoproliferative disorder.
